# Assessing Visual Avoidance of Faces During Real-Life Social Stress in Children with Social Anxiety Disorder: A Mobile Eye-Tracking Study

**DOI:** 10.1007/s10578-022-01383-y

**Published:** 2022-06-16

**Authors:** Leonie Rabea Lidle, Julian Schmitz

**Affiliations:** 1 Department for Clinical Child and Adolescent Psychology, Institute of Psychology, Leipzig University, Neumarkt 9–19, 04109 Leipzig, Germany; 2 Leipzig Research Centre for Early Child Development, Leipzig University, Leipzig, Germany

**Keywords:** Social anxiety disorder, Children, Visual attention, Attentional bias, Avoidance

## Abstract

This study measured visual attention (fixation count, dwell time) during two real-life social stress tasks using mobile eye-tracking glasses in children (9–13 years) diagnosed with social anxiety disorder (SAD; *n* = 25) and a healthy control group (HC; *n* = 30). The influence of state anxiety on attention allocation and negative self-evaluation biases regarding gaze behavior were also examined. Compared to the HC group, children with SAD showed visual avoidance (i.e., fewer fixations) of the faces of interaction partners during the second social stress task. While visual avoidance in HC children decreased with declining state anxiety from the first to the second social stress task, no such effect was found in children with SAD. A negative self-evaluation bias regarding gaze behavior in children with SAD was not found. In sum, measuring visual attention during real-life social situations may help enhance our understanding of social attention in childhood SAD.

**Supplementary Information:**

The online version contains supplementary material available at 10.1007/s10578-022-01383-y.

## Introduction

Social anxiety disorder (SAD), characterized by a persistent and excessive fear of negative evaluation by others, is one of the most common mental disorders in childhood [1]. SAD typically emerges early in life, with a median age of onset in late childhood to midteens [2]. Commonly diagnosed among clinically anxious children seeking help (e.g., 45%) [3], SAD constitutes a significant burden for healthcare. Yet, children and adolescents with SAD are less likely to respond favorably to cognitive behavioral therapy (CBT) compared to children with other anxiety disorders [4], highlighting the need for experimental research on maintenance factors of social anxiety in childhood.

Cognitive models of SAD (e.g. [5, 6]) propose that the disorder is maintained by biased cognitive processing of social information, such as abnormalities in visual attention during social situations (e.g., direct eye contact) and negatively distorted self-evaluations. Specifically, the attentional avoidance of social information, such as eye gaze and human faces in general, is considered an important feature of the disorder. As facial expressions and specific facial areas, for example, the eye region, convey crucial information for social interactions (e.g., emotional state of others) [7], socially anxious individuals lose important social information that could correct negative social self-beliefs and schemata (e.g., an audience member looking more encouraging over the course of a presentation). Consequently, the visual avoidance of threat-related stimuli may prevent habituation and anxiety extinction. The visual avoidance of faces and eye contact may further be misinterpreted as a sign of disinterest by others, leading to an increased risk of being perceived as less likeable by peers [8]. Besides the visual avoidance of social information, cognitive models assume that individuals with SAD additionally show a negatively biased self-perception of their own social performance, which also includes parameters of social attention (e.g., self-ratings of eye contact), which in turn further strengthen negative self-beliefs.

Although a recent meta-analysis concluded that adults with SAD exhibit visual avoidance of faces across different social situations [9], to date, less is known about visual attention to faces and facial features in childhood SAD. Given that the brain circuits contributing to attentional deployment regarding emotional stimuli—and more particularly anxiety-linked attention—change with maturation, it is unlikely that adult findings can simply be extrapolated to child populations (e.g., [10]).

In this vein, eye-tracking methodology is particularly useful to examine attentional biases during visual face processing because it allows for spatially precise and noninvasive measurement of visual attention allocation. However, previous eye-tracking studies in children and adolescents diagnosed with SAD showed mixed results regarding attentional biases toward faces. While some found evidence of the visual avoidance of emotional faces in children diagnosed with SAD aged 9 to 13 years at later processing phases during the passive viewing of face stimuli [11, 12] and a positive association between the severity of anxiety symptoms and the avoidance of eye contact in a subclinical sample of children with mixed anxiety symptoms during fear acquisition [13], others failed to find an association between social anxiety and visual avoidance of faces in children diagnosed with SAD aged 8 to 12 years (e.g., [14]).

In addition to the inconsistent research findings regarding the visual avoidance of faces in children with SAD, there are several aspects of the existing research that suggest further study is warranted. First, studies in children diagnosed with SAD predominantly used static social stimuli such as pictures of faces (e.g., [12]), which may allow the assessment of attentional processes under highly controlled conditions but is associated with a severely limited ecological validity [15]. Research has also suggested that visual attention patterns differ significantly between real-life social interactions and static computer-screen-based presentations (e.g., [16]). In this context, mobile eye-tracking methodology (e.g., eye-tracking glasses) enables the assessment of attentional patterns in ecologically valid contexts such as real-life social situations (e.g., [15]). In adults, studies assessing gaze behavior during real-life social interaction tasks in nonclinical undergraduate samples [17, 18] and during a speech task implementing a virtual audience in a clinical sample with SAD [19] demonstrated an association between social anxiety and reduced visual attention to faces. However, research on visual attention to faces in children diagnosed with SAD during real-life social situations (e.g., during speech tasks) using mobile eye-tracking technology is still missing.

Second, theoretically important moderating influences on visual avoidance in childhood SAD remain unclear. For example, Seefeldt et al. [14] and Schmidtendorf et al. [12] demonstrated a visual attentional bias toward emotional faces (i.e., initial hypervigilance regarding angry compared to neutral faces) in children diagnosed with SAD aged 8 to 13 years compared to a control group only after a social stress induction, suggesting that state anxiety may have a significant influence on visual attention to faces in children diagnosed with SAD. However, as these studies implemented a threat induction condition (i.e., announcement of a speech task) but did not measure visual attention directly during a real-life social situation, associations between subjectively experienced state anxiety and visual attention remain uncertain.

Third, cognitive models of SAD (e.g., [5]) assume that individuals with SAD can show not only an objective social performance deficit (e.g., avoidance of eye contact) but also negatively distorted perceptions of their own performance during social situations. This implies that negative self-evaluations not only reflect actual social skill deficits but are *further* negatively biased. While a negative evaluation bias was shown regarding *global* measures of social performance in high compared to low socially anxious children (e.g., [20, 21]; for an exception see, e.g., [22]), self-evaluation biases specifically regarding gaze behavior based on objective eye-tracking measures have not yet been investigated in children diagnosed with SAD.

Taking the limitations of previous research as a starting point, the present study examined visual attention in children diagnosed with SAD compared to a healthy control (HC) group during a real-life social stress task using mobile eye-tracking technology while measuring the possible moderating influence of state anxiety and self-evaluations of social attention. We first hypothesized (Hypothesis 1) that compared to the HC group, children diagnosed with SAD would show reduced attention to the faces of their interaction partners during a social stress task as indicated by (a) a reduced number of fixations and (b) a reduced total dwell time on the faces of interaction partners throughout the social stress task (e.g., [19]). We further hypothesized (Hypothesis 2) that higher state anxiety during the social stress task would be associated with reduced visual attention as indicated by (a) a lower number of fixations and (b) a lower total dwell time on the faces of interaction partners during the social stress task in both groups (e.g., [12]). In addition, we hypothesized (Hypothesis 3a) that children diagnosed with SAD would rate their subjectively perceived amount of gaze behavior directed toward interaction partners during the social stress tasks as significantly lower than the HC group’s ratings. We further predicted a negative self-evaluation bias regarding gaze behavior in children diagnosed with SAD (Hypothesis 3b), that is, that only in the HC group—and not in children diagnosed with SAD—would self-ratings of gaze behavior correspond to objective measures of gaze behavior (number of fixations, dwell time; e.g., [20]).

## Method

### Participants

Participant characteristics are found in Table 1. Children aged 9 to 13 years were recruited from the community through information letters, flyers, and advertisements. In compensation for participation, families were offered a child appropriate voucher (30€) and feedback regarding the diagnostic information assembled during study participation. Following a telephone screening, eligible families were invited to take the Kinder-DIPS [23], a modified version of the Anxiety Disorders Interview Schedule for Children—Revised [24]. Inclusion criterion for the group with SAD was a primary diagnosis of SAD according to the fifth edition of the *Diagnostic and Statistical Manual of Mental Disorders* (*DSM-5*) [25]. Exclusion criteria were health conditions that could alter psychophysiological assessment (e.g., asthma, cardiac arrhythmia, treatment with methylphenidate), and past or current psychological treatment. The age- and gender-matched HC group did not have any lifetime diagnosis of a mental disorder. Of the initially included 62 participants, seven children diagnosed with SAD had to be excluded (irregularities in the study procedure [*n* = 2], low eye-tracking data quality [*n* = 5]). All participants spoke German fluently and had normal or corrected-to-normal vision.

**Table 1:** Participant characteristics

Characteristic	Group		Statistics
	Children with social anxiety disorder	Healthy controls	
Sample size (*n*)	25	30	
Mean age (*SD*), in years	11.8 (1.11)	11.6 (1.12)	*t*(53) = − 0.71, *p* = 0.482
Female (%)	64.0	60.0	χ^2^(1) = 0.09, *p* = 0.761
Mean SASC-R (*SD*)	51.5 (13.7)	28.4 (6.16)	*t*(32.0) = − 7.80, *p* < 0.001
Mean CDI (*SD*)	17.5 (9.12)	5.90 (3.50)	*t*(29.9) = − 6.02, *p* < 0.001
School (%)			
Grammar school (%)	88.0	76.7	χ^2^(1) = 1.18, *p* = 0.278
Comprehensive school (%)	8.00	10.0	χ^2^(1) = 0.07, *p* = 0.797
Primary school (%)	4.00	6.67	χ^2^(1) = 0.19, *p* = 0.665
Other (%)	0.00	6.67	χ^2^(1) = 1.73, *p* = 0.189
Parental marital status (% separated)	36.0	3.33	χ^2^(1) = 9.78, *p* = 0.002
Comorbid diagnoses		NA	
Specific phobia (%)	36.0		
General anxiety disorder (%)	16.0		
Child separation anxiety disorder (%)	8.00		
Elective mutism (%)	4.00		
Sleeping disorders (%)	4.00		
Depressive disorders (%)	4.00		
Dyslexia (%)	4.00		

*CDI* Children’s Depression Inventory; *SASC*-*R* Social Anxiety Scale for Children—Revised; *NA* not available for sample

### Ethical Considerations

The study was granted ethical approval by Leipzig University’s Research Ethics Committee. Parents and children were both provided with written and verbal information about the study. Written parental consent and child assent were both required to participate in the study. All participants received a child-appropriate voucher (€30) as compensation and children in the clinical group were offered treatment in the department’s outpatient clinic.

### Experimental Procedure

The study employed a 2 × 2 repeated measures design, consisting of two experimental groups (SAD vs. HC) and two repeated measurement points (Session 1 [S1] and Session 2 [S2] of the social stress task).^1^ After participating in our diagnostic session, which included the Kinder-DIPS [23] and a self-report-measure of trait social anxiety (Social Anxiety Scale for Children—Revised [26]), eligible children were reinvited to a 90-min experimental session consisting of two separate social stress tasks. Each session of the social stress task consisted of a 4-min social performance situation during which the participants answered questions about a previously heard short story in front of two unknown female observers (i.e. interaction partners) [27, 28]. The interaction partners alternated asking four standardized questions at defined time intervals of 1 min. If children stopped talking before the 1 min interval was up, the interaction partners prompted them to continue talking. All interaction partners were trained to offer eye contact continuously during the social stress tasks and to show a neutral but friendly attitude toward the participants. Children were instructed to do their best because the interaction partners and peers would later rate their performance based on video recordings [28, 29]. Both sessions of the social stress task were separated by a 10-min break during which children were offered to play a well-known game of cards (UNO®).

### Measures

#### Structured Diagnostic Interview with Children and Parents

Children were assigned diagnoses based on the Kinder-DIPS [23], which was separately administered to each child and a parent. This instrument enables standardized clinical assessments of lifetime diagnoses, current and past, according to the criteria of the 10th revision of the *International Statistical Classification of Diseases and Related Health Problems* [30] and the *DSM-5* [25]. The Kinder-DIPS has good validity and reliability for Axis I disorders, and the kappa values of the Kinder-DIPS lifetime diagnoses (child report: 0.90–0.98, parent report: 0.88–0.97) are high to very high [31].

#### Social Anxiety Scale for Children—Revised

Social anxiety symptoms were measured using the Social Anxiety Scale for Children—Revised [26]. The 22-item self-report questionnaire is validated for children and adolescents aged 8 to 16 years and can be grouped into two subscales measuring fear of negative evaluation (e.g., “I worry about what other kids think of me”) and social anxiety and distress (e.g., “I feel nervous when I talk to kids I don’t know very well”). Children respond to all items on a Likert scale of 1 (*not at all*) to 5 (*all of the time*). Total scores range from 18 to 90. The German version of the questionnaire [32] shows satisfactory retest reliability (after 2 weeks: 0.74 ≤ r_tt_ ≤ 0.84) [33] and internal consistency (*α* = 0.63–0.83) in community samples aged 7 to 16 years [32, 33]. The internal consistency in the current sample was excellent (α = 0.95).

#### Children’s Depression Inventory

The Children’s Depression Inventory (CDI) [34] is a self-report questionnaire measuring depressive symptoms according to the *DSM-5* in children aged 8 to 16 years. The German version [35] consists of 29 items and total scores can range from 0 to 58. For each item, children are asked to choose from three statements the one that best describes the way they have been feeling lately (e.g., “I am sad once in a while” [0], “I am sad many times” [1], “I am sad all the time” [2]). The CDI reliably differentiates between children with and without depression at age 9 to 12 years (sensitivity: 91.7%, specificity: 81.9%) [36] and has shown adequate internal consistencies in children aged 8 to 16 years (α = 0.82–0.87) [35, 36]. The internal consistency in the present sample was excellent (α = 0.93).

#### State Anxiety Ratings

Participants rated the subjective anxiety experienced during the social stress tasks (“How scared were you while answering questions about the story in front of the two observers?”) on a visual analogue scale of 0 (*no anxiety*) to 10 (*extreme anxiety*) immediately after each session of the social stress task [27, 28].

#### Self-rating of Gaze Behavior

Participants rated their gaze behavior directed toward the interaction partners during the social stress tasks on a 4-point scale of 1 (*very much*) to 4 (*not very much*) immediately after completing each session of the social stress task (“How much did you look at the person you were talking to?”). The item is part of the revised Performance Questionnaire—Child version [20], a nine-item questionnaire designed to measure social performance during a speech task. For the current analysis, the item was reversed so that higher scores indicate a higher self-rating of gaze behavior.

### Eye-Tracking System and Coding

A mobile eye-tracking device (Eye Tracking Glasses 2w, ETG 2.0, SensoMotoric Instruments [SMI], Teltow, Germany), worn like a normal pair of glasses, continuously tracked participants attention to the interaction partners during the social stress tasks at a 60-Hz binocular sampling rate. A high-definition camera captured the participants’ visual field and the wearer’s gaze point was mapped into a scene video. SMI iViewETG software (version 2.7) was used for a three-point calibration at a distance of 1.20 m and for recording. The calibration was conducted immediately before and after each social stress task to allow the monitoring of slippage during the tracking phase [37]. The tracker has a reported gaze position accuracy of 0.5° and precision of 0.1°, and the gaze-tracking range extends 80° horizontally and 60° vertically.

Eye-tracking data were processed and coded using SMI’s standard software (BeGaze 3.7). Gaze events were automatically coded using the built-in event detector, a velocity-based algorithm based on the thresholds of α_def_ = 100°/s, α_min_ = 8°/s, β = 5 and γ = 0.5 (for a detailed description, see BeGaze Manual). Fixations then were manually mapped by two independent raters, who were blind to group status, onto an abstract template showing all areas of interest (AOIs). Specifically, an AOI was created around the entire face of the interaction partners (“face AOI”; see Supplement A). Fixations on the faces of both interaction partners were mapped onto a single template. Our main outcome parameters were the fixation count, defined as the number of fixations inside the face AOI, and the dwell time, defined as the sum of durations of all fixations and saccades in the face AOI measured in milliseconds. To ensure interrater reliability, 45.96% of the sampled videos were coded by both raters. Excellent interrater reliability was found for fixation count (Session 1/2: ICC = 0.983/0.964) and dwell time (Session 1/2: ICC = 0.988/0.961). Five children diagnosed with SAD had to be excluded from data analysis because of a lack of adequate eye-tracking calibration. No group differences emerged between excluded and included children diagnosed with SAD regarding trait social anxiety, depression scores, or gender, all *p*s ≥ 0.273, but excluded children were on average older than children showing good tracking quality, *t*(30) = − 2.076, *p* = 0.047.

### Data Analysis

Statistical analyses were conducted using the open statistics software R [38]. Hypothesis 1a and b and Hypothesis 3a were evaluated via mixed linear models (MLMs) to account for the hierarchical nature of the data. The mixed model packages lme4 [39] and lmerTest [40] were used. As proposed by Luke [41], MLMs were fitted using restricted maximum likelihood, and *p* values were derived with Kenward–Roger approximation to account for small sample sizes. All MLMs were fitted with one between-subjects factor, group (SAD, HC), and one within-subject factor, session (S1, S2), and all possible interaction terms as fixed effects. All models included a random intercept to control for subject effects, and model-based follow-up analyses are reported. To analyze Hypothesis 2a and b and Hypothesis 3b, stepwise multiple regression models were conducted. Regression models were built separately for both experimental groups and included both sessions in long format. To analyze Hypothesis 2a and b, first trait social anxiety and second subjective state anxiety during the social stress task were included as predictors of gaze behavior (fixation count, dwell time) in the regression model. Regarding Hypothesis 3b, we successively entered first trait social anxiety, second subjective state anxiety during the social stress task, and third the respective measure of gaze behavior (fixation count, dwell time) in the regression model as predictors of self-ratings of gaze behavior directed toward the interaction partners. Because of possible developmental influences [42] and research findings indicating an influence of gender on visual face processing [11], age and gender were included as control variables in all regression models. Adjusted *R*^2^ and the Akaike information criterion are reported. The level of significance was set at α = 0.05 for all statistical analyses and Cohen’s *d* or η_p_^2^ are reported as measures of effect size.

## Results

### Preliminary Analyses: Anxiety Levels During the Social Stress Tasks and Time Course of Attention

The MLM comparing the subjective anxiety evoked during the social stress tasks between children diagnosed with SAD and the HC group revealed significant main effects of group, *F*(1, 53) = 61.561, *p* < 0.001, η_p_^2^ = 0.54, 95% confidence interval (CI) [0.35, 0.67], and session, *F*(1, 53) = 59.852, *p* < 0.001, η_p_^2^ = 0.53, 95% CI [0.34, 0.66]. The interaction effect of Group × Session was not significant, *F*(1, 53) = 0.495, *p* = 0.485, η_p_^2^ < 0.01, 95% CI [0.00, 0.12]. Model-based follow-up analyses revealed that children diagnosed with SAD reported significantly higher levels of subjective anxiety than the HC group during S1, *t*(67.40) = 7.599, *p* < 0.001, *d* = 2.16, 95% CI [1.48, 2.85], and S2, *t*(67.40) = 7.108, *p* < 0.001, *d* = 1.84, 95% CI [1.19, 2.49]. Both groups reported significantly lower subjective anxiety levels during S2 compared to S1: group with SAD, *t*(53.0) = − 5.714, *p* < 0.001, *d* = 0.67, 95% CI [0.09, 1.26]; HC group, *t*(53.0) = − 5.216, *p* < 0.001, *d* = 0.81, 95% CI [0.28, 1.35]; also see Table 2.

**Table 2:** Descriptive statistics of the main outcome variables fixation count and dwell time, self-ratings of gaze behavior, and subjective anxiety during the social stress task by group and session

	Children with social anxiety disorder group	Healthy control group
Fixation count		
Session 1	159.40 (113.09)^a^	180.90 (106.59)^b^
Session 2	143.84 (114.95)^a^	211.87 (134.48)^b^
Dwell time (ms)		
Session 1	65,781.84 (54,900.97)^a^	66,465.96 (42,901.93)^b^
Session 2	59,556.93 (55,069.51)^a^	80,721.98 (51,732.63)^b^
Self-rating of gaze behavior^c^		
Session 1	2.40 (1.08)^a^	2.90 (1.09)^b^
Session 2	2.28 (0.89)^a^	3.33 (0.76)^b^
Subjective anxiety^d^		
Session 1	7.48 (2.18)^a^	2.83 (2.12)^b^
Session 2	5.68 (3.09)^a^	1.33 (1.52)^b^

Standard deviation is indicated in brackets

^a^Based on a sample size of *n* = 25

^b^Based on a sample size of *n* = 30

^c^Children’s retrospective self-rating of gaze behavior on a scale of 1 to 4; higher scores indicate higher subjective ratings of gaze behavior

^d^Children’s retrospective self-rating of anxiety on a scale of 0 to 10; higher scores indicate higher subjective anxiety

To analyze if gaze behavior changed across the time span of our social stress tasks, we divided the 4-min social stress tasks into four 1-min time segments. The analyzed MLMs included group (SAD, HC) as between-subjects factor, time (Minute 1–4) and session (S1, S2) as within-subject factors and all possible interaction terms as fixed effects. Including the repeated measures showed no significant Group × Time interactions, all *F*s(3, 371) ≤ 0.446, all *p*s ≥ 0.720, or Session × Time interactions, all *F*s(3, 371) ≤ 1.074, all *p*s ≥ 0.360, when predicting the gaze indices, indicating that changes in gaze behavior across time were independent of group status and session. Follow up analysis indicated that significant main effects of time indicated a lower fixation count and dwell time in Minute 1 compared to Minute 4 across all participants (all *p*s ≤ 0.003): fixation count, *F*(3, 379) = 3.310, *p* = 0.020, η_p_^2^ = 0.03, 95% CI [0.00, 0.06]; dwell time, *F*(3, 379) = 3.053, *p* = 0.028, η_p_^2^ = 0.02, 95% CI [0.00, 0.06]. All other time comparisons did not reach statistical significance, all *p*s ≥ 0.052. We thus averaged our outcome parameters across the 4-min time span of the social stress tasks and based the following analyses on the averaged values. As significant Group × Session interactions regarding both gaze parameters indicated a significant influence of session on gaze behavior [fixation count: *F*(1, 371) = 10.652, *p* = 0.001; dwell time: *F*(1, 371) = 13.928, *p* < 0.001], Session was included as a predictor in the analysis of Hypothesis 1a and b and Hypothesis 3a.^2^

### Hypothesis 1a and b: Visual Attention to Faces During the Social Stress Tasks

The MLM regarding Hypothesis 1a revealed a significant interaction of Group × Session, *F*(1, 53) = 4.110, *p* = 0.048, η_p_^2^ = 0.07, 95% CI [0.00, 0.23]. The main effects of group, *F*(1, 53) = 2.249, *p* = 0.140, η_p_^2^ = 0.04, 95% CI [0.00, 0.19], and session, *F*(1, 53) = 0.451, *p* = 0.505, η_p_^2^ < 0.01, 95% CI [0.00, 0.11], were not significant. Model-based follow-up analyses indicated that the two groups did not differ concerning the fixation count during S1, *t*(68.33) = − 0.672, *p* = 0.504, *d* = 0.20, 95% CI [− 0.35, 0.74], while children diagnosed with SAD looked significantly less at the faces of their interaction partners than the HC group during S2, *t*(68.33) = − 2.127, *p* = 0.037, *d* = 0.54, 95% CI [− 0.01, 1.09]. While children diagnosed with SAD did not differ in their fixation count between S1 and S2, *t*(53.0) = − 0.918, *p* = 0.363, *d* = 0.14, 95% CI [− 0.43, 0.71], there was a trend for HC children to look more often at the faces of their interaction partners during S2 compared to S1, *t*(53.0) = 2.001, *p* = 0.051, *d* = 0.26, 95% CI [− 0.26, 0.77].

The analysis regarding Hypothesis 1b showed a significant interaction effect of Group × Session, *F*(1, 53) = 5.268, *p* = 0.026, η_p_^2^ = 0.09, 95% CI [0.00, 0.26], while the main effects of group, *F*(1, 53) = 0.698, *p* = 0.407, η_p_^2^ = 0.01, 95% CI [0.00, 0.13], and session, *F*(1, 53) = 0.810, *p* = 0.372, η_p_^2^ = 0.02, 95% CI [0.00, 0.13], were not significant. Model-based follow-up analyses showed that the two groups did not differ regarding dwell time on the face AOI of their interaction partners during S1, *t*(65.17) = − 0.049, *p* = 0.961, *d* = 0.01, 95% CI [− 0.53, 0.56], and S2, *t*(65.17) = − 1.531, *p* = 0.131, *d* = 0.40, 95% CI [− 0.15, 0.95]. While children diagnosed with SAD did not differ in dwell time on the face AOI of their interaction partners between S1 and S2, *t*(53.0) = − 0.945, *p* = 0.349, *d* = 0.11, 95% CI [− 0.15, 0.37], HC children showed a significantly higher dwell time during S2 compared to S1, *t*(53.0) = 2.370, *p* = 0.022, *d* = 0.29, 95% CI [0.06, 0.52].

### Hypothesis 2a and b: Influence of State Anxiety on Attention Allocation

In the HC group, subjective anxiety experienced during the two sessions of the social stress task significantly predicted the fixation count (β = − 0.342, *p* = 0.014) in the final regression model including trait social anxiety and subjective state anxiety during the social stress tasks, while controlling for age and gender, *F*(4, 55) = 2.945, *p* = 0.028, adjusted *R*^2^ = 0.117 (see Table 3). Furthermore, state anxiety was the only significant predictor of dwell time on the face AOI of the interaction partners (β = − 0.398, *p* = 0.005) in the equivalent regression model, *F*(4, 55) = 2.617, *p* = 0.045, adjusted *R*^2^ = 0.099 (see Table 4). Higher subjective anxiety experienced during the social stress tasks was thus associated with a lower fixation count and dwell time in the HC group. In the group with SAD, the analyzed regression models regarding the predictive validity of state anxiety on fixation count and dwell time did not reach statistical significance, fixation count: *F*(4, 45) = 0.808, *p* = 0.527, adjusted *R*^2^ = − 0.016; dwell time: *F*(4, 45) = 0.682, *p* = 0.608, adjusted *R*^2^ = − 0.027 (see Tables 3 and 4).

**Table 3:** Regression coefficients (β) explaining variance in the count of fixations on the facial region of the interaction partners during the social stress tasks in the group with social anxiety disorder and in the healthy control group

Predictor	Step			
	I	II	III	IV
Group with social anxiety disorder				
Gender	0.078	0.095	0.069	0.096
Age		0.093	0.084	0.087
SASC-R			0.145	0.229
State anxiety^a^				− 0.203
Adjusted *R*^2^	− 0.015	− 0.028	− 0.028	− 0.016
*R*^2^ change	− 0.015	− 0.013	− 0.001	0.013
*F* change	0.292	0.394	0.962	1.566
AIC	619.43	621.02	621.02	622.27
Healthy control group				
Gender	0.085	0.133	0.168	0.093
Age		0.198	0.224	0.172
SASC-R			− 0.194	− 0.057
State anxiety^a^				− 0.342*
Adjusted *R*^2^	− 0.010	0.010	0.031	0.117
*R*^2^ change	− 0.010	0.020	0.020	0.086
*F* change	0.418	2.196	2.193	6.436*
AIC	750.64	750.37	750.07	745.43

Group with social anxiety disorder: minimal tolerance = 0.788, maximal variance inflation factor = 1.269. Healthy control group: minimal tolerance = 0.809, maximal variance inflation factor = 1.236

*AIC* Akaike information criterion, *SASC*-*R* Social Anxiety Scale for Children—Revised

*p < 0.05

^a^Subjective anxiety during social stress task

**Table 4:** Regression coefficients (β) explaining variance in time spent looking at the facial region of the interaction partners (dwell time, milliseconds) during the social stress tasks in the group with social anxiety disorder and in the healthy control group

Predictor	Step			
	I	II	III	IV
Group with social anxiety disorder				
Gender	0.045	0.074	0.051	0.070
Age		0.154	0.147	0.149
SASC-R			.128	0.188
State anxiety^a^				− 0.144
Adjusted *R*^2^	− 0.019	− 0.017	− 0.022	− 0.027
*R*^2^ change	− 0.019	0.002	− 0.005	− 0.005
*F* change	0.097	1.103	0.762	0.780
AIC	1237.40	1238.24	1239.42	1240.56
Healthy control group				
Gender	0.078	0.095	0.120	0.033
Age		0.070	0.089	0.028
SASC-R			− 0.140	0.020
State anxiety^a^				− 0.398**
Adjusted *R*^2^	− 0.011	− 0.024	− 0.023	0.099
*R*^2^ change	− 0.011	− 0.013	0.002	0.121
*F* change	0.354	0.263	1.084	8.541**
AIC	1467.53	1469.25	1470.10	1463.44

Group with social anxiety disorder: minimal tolerance = 0.788, maximal variance inflation factor = 1.269. Healthy control group: minimal tolerance = 0.809, maximal variance inflation factor = 1.236

*AIC* Akaike information criterion, *SASC*-*R* Social Anxiety Scale for Children—Revised

**p < 0.01

^a^Subjective anxiety during social stress task

### Hypothesis 3a: Self-evaluation of Gaze Behavior

The MLM regarding Hypothesis 3a revealed a significant main effect of group, *F*(1, 53) = 12.043, *p* = 0.001, η_p_^2^ = 0.19, 95% CI [0.04, 0.37], and a significant interaction of Group × Session, *F*(1, 53) = 4.255, *p* = 0.044, η_p_^2^ = 0.07, 95% CI [0.00, 0.24]. The main effect of session was not significant, *F*(1, 53) = 1.364, *p* = 0.248, η_p_^2^ = 0.03, 95% CI [0.00, 0.16]. Model-based follow-up analyses showed a trend for children diagnosed with SAD to report lower self-ratings of gaze behavior than the HC group in S1, *t*(86.72) = − 1.916, *p* = 0.059, *d* = 0.46, 95% CI [− 0.09, 1.01], and significantly lower self-ratings of gaze behavior than the HC group in S2, *t*(86.72) = − 4.037, *p* < 0.001, *d* = 1.28, 95% CI [0.69, 1.88]. The self-ratings of gaze behavior did not differ significantly between S1 and S2 in children diagnosed with SAD, *t*(53.0) = − 0.606, *p* = 0.547, *d* = 0.12, 95% CI [− 0.45, 0.69], whereas HC children reported significantly higher levels of gaze behavior during S2 compared to S1, *t*(53.0) = 2.396, *p* = 0.020, *d* = 0.46, 95% CI [− 0.06, 0.98].

### Hypothesis 3b: Negative Self-evaluation Bias Regarding Gaze Behavior

In the HC group, the fixation count was the only significant predictor of self-ratings of gaze behavior directed toward the interaction partners (β = 0.291, *p* = 0.029) in the final regression model including trait social anxiety, state anxiety experienced during the social stress tasks, and the fixation count, while controlling for age and gender, *F*(5, 54) = 3.578, *p* = 0.007, adjusted *R*^2^ = 0.179 (see Table 5). Regarding the predictive validity of dwell time, in the final regression model, age (β = 0.251, *p* = 0.049) and dwell time (β = 0.287, *p* = 0.030) were significant predictors of self-ratings of gaze behavior, *F*(5, 54) = 3.567, *p* = 0.007, adjusted *R*^2^ = 0.179. A higher fixation count and a higher dwell time on the face AOI of the interaction partners thus were associated with higher self-ratings of gaze behavior in the HC group, while age was associated with higher self-ratings of gaze behavior only regarding the dwell time.

**Table 5:** Regression coefficients (β) explaining variance in the self-ratings of gaze behavior directed toward the interaction partners during the social stress tasks in the group with social anxiety disorder and in the healthy control group

Predictor	Step					
	I	II	III	IV	V^a^ Fixation count	V^b^ Dwell time
Group with social anxiety disorder						
Gender	− 0.252	− 0.291*	− 0.316*	− 0.310*	− 0.367**	− 0.352**
Age		− 0.207	− 0.215	− 0.214	− 0.266*	− 0.303*
SASC-R			0.133	0.150	0.013	0.038
State anxiety^c^				− 0.040	0.081	0.046
Gaze parameter					0.594***	0.593***
Adjusted *R*^2^	0.044	0.067	0.065	0.045	0.390	0.394
*R*^2^ change	0.044	0.023	− 0.002	− 0.019	0.345	0.348
*F* change	3.26	2.18	0.90	0.06	26.45***	26.82***
AIC	141.75	141.49	142.52	144.45	122.92	122.65
Healthy control group						
Gender	0.064	0.129	0.172	0.115	0.088	0.105
Age		0.267*	0.299*	0.259*	0.209	0.251*
SASC-R			− 0.233	− 0.129	− 0.112	− 0.134
State anxiety^c^				− 0.260	− 0.161	− 0.146
Gaze parameter					0.291*	0.287*
Adjusted *R*^2^	− 0.013	0.039	0.076	0.119	0.179	0.179
*R*^2^ change	− 0.013	0.052	0.038	0.043	0.060	0.059
*F* change	0.24	4.11*	3.33	3.73	5.01*	4.97*
AIC	169.90	167.72	166.26	164.31	160.99	161.03

*AIC* Akaike information criterion, *SASC*-*R* Social Anxiety Scale for Children—Revised

*p < 0.05, **p < 0.01, ***p < 0.001

^a^Regression model including the fixation count as predictor. Group with social anxiety disorder: minimal tolerance = 0.762, maximal variance inflation factor = 1.313. Healthy control group: minimal tolerance = 0.736, maximal variance inflation factor = 1.359

^b^Regression model including dwell time (ms) as predictor. Group with social anxiety disorder: minimal tolerance = 0.773, maximal variance inflation factor = 1.294. Healthy control group: minimal tolerance = 0.712, maximal variance inflation factor = 1.405

^c^Subjective anxiety during social stress task

In the group with SAD, the analyzed regression model regarding Hypothesis 3b showed that gender (β = − 0.367, *p* = 0.003), age (β = − 0.266, *p* = 0.024), and fixation count (β = 0.594, *p* < 0.001) significantly predicted self-ratings of gaze behavior, *F*(5, 44) = 7.272, *p* < 0.001, adjusted *R*^2^ = 0.390 (see Table 5). A similar pattern was shown regarding dwell time, as gender (β = − 0.352, *p* = 0.004), age (β = − 0.303, *p* = 0.011), and dwell time (β = 0.593, *p* < 0.001) significantly predicted self-ratings of gaze behavior in the final regression model, *F*(5, 44) = 7.359, *p* < 0.001, adjusted *R*^2^ = 0.394. While a higher fixation count and a higher dwell time predicted higher self-ratings of gaze behavior in children diagnosed with SAD, age as well as female gender were associated with lower self-ratings of gaze behavior.

## Discussion

The present study investigated visual attention to faces in children diagnosed with SAD compared to a group of HC children. We assessed gaze behavior using mobile eye-tracking glasses during a real-life social performance situation in our laboratory, extending the external and ecological validity of previous studies that predominantly implemented static face stimuli presented for short time periods (e.g., [11, 12]). We further examined if state anxiety experienced during the social performance situation was associated with reduced visual attention to faces in all children and if children diagnosed with SAD showed a negative evaluation bias regarding gaze behavior, as has been proposed by cognitive models (e.g., [5]).

Partly confirming our first hypothesis, children diagnosed with SAD showed reduced visual attention to (i.e., fewer fixations on) the faces of their interaction partners compared to the control group during the second session of the social stress task but not during the first. A similar trend emerged for our second outcome parameter, dwell time. Our results partly replicate findings in adults diagnosed with SAD showing a tendency to avoid visual attention to a virtual audience during a speech task [19]. A possible explanation for the unexpected similar gaze patterns in the two experimental groups during the first session of the social stress task may be a tendency to avoid visual attention to faces in high social stress conditions in all children. However, while children in the control group seemed to habituate to the social stress task, as indicated by higher visual attention to the interaction partners along with reduced anxiety levels during the second session of the social stress task, children diagnosed with SAD showed stable gaze behavior across both sessions. Further support for this interpretation comes from positive relations between state anxiety and visual attention parameters in the HC group but not in the group with SAD (cf. Hypothesis 2). Hence, children with SAD may show a stable dysfunctional gaze pattern of visual avoidance in real-life situations unrelated to state anxiety. A possible reason for this insensitivity of gaze parameters to situational stress levels may be the use of safety behavior strategies in children diagnosed with SAD aimed at reducing subjective anxiety during the anxiety-provoking social stress task (e.g., attention allocation toward less threatening, neutral situational aspects) [43, 44]. If these strategies are habitually implemented, they may prevent children diagnosed with SAD from flexibly adjusting their gaze behavior over repeated exposures. This may further explain why children with SAD are less responsive to CBT than children with other anxiety disorders [4]. To validate this hypothesis, we encourage future studies to include measures of safety behaviors.

Contrary to our second hypothesis, only in the HC group and not in children diagnosed with SAD did subjective anxiety during the social stress tasks statistically predict lower visual attention, measured by both the fixation count and the dwell time, to the faces of the interaction partners. By contrast, theoretical models (e.g., [5]) proposed enhanced attentional biases under conditions of social-evaluative threat and studies in children with SAD have demonstrated visual attentional biases only after a social stress induction [12, 14]. Several explanations are conceivable for these conflicting findings. As previous studies focused primarily on early visual attention processes (e.g., 3000–5000 ms) [12, 14] and thus predominantly assessed automatic bottom-up driven visual attention, results might not be applicable to longer exposure durations in real-life contexts. Deliberate top-down processes such as the discussed use of safety behaviors may therefore explain the missing association between state anxiety and visual attention in our sample. Gaze behavior may also have been effortfully controlled because of clear behavioral demands associated with the implemented social stress task, thus reducing the proposed association between state social anxiety and gaze behavior, especially in the children diagnosed with SAD who may have been more susceptible to social desirability effects.

Regarding children’s self-evaluation of gaze behavior (Hypothesis 3a), children diagnosed with SAD reported lower levels of visual attention to the interaction partners only during the second and not the first session of the social stress task when compared to HC children. This effect was mainly driven by HC children reporting significantly higher levels of visual attention to the interaction partners during the second compared to the first session of the social stress task, while children with SAD reported comparable visual attention during both sessions. Interestingly, these self-ratings of gaze behavior were significantly associated with objective gaze parameters, fixation count and dwell time, in both groups. Consistent with several research findings (e.g., [22, 45]), a negative evaluation bias regarding self-rated gaze behavior thus was not found in our group with SAD. The statistically high accordance found between children’s self-ratings of gaze behavior and the objectively measured eye-tracking parameters in children with SAD may be explained by a self-focused processing mode, as proposed by Clark and Wells [5]. The authors postulated that socially anxious individuals enter anxiety-provoking social situations focusing on their own behavior and how they may look in others’ eyes more than on external aspects of the situation, for example, reactions of interaction partners. The children diagnosed with SAD thus may have monitored their gaze behavior more closely, especially as the mobile eye-tracking glasses likely heightened awareness of this performance aspect. Furthermore, while in the group with SAD, higher age and female gender were associated with lower self-ratings of gaze behavior directed toward the interaction partners, older children in the HC group rated their gaze behavior more positively. The opposing associations of age and self-ratings of gaze behavior in both experimental groups may indicate that while non-anxious children develop a more positive self-concept regarding their social performance over time, children with SAD develop a realistic and possibly more critical self-concept regarding social performance parameters [45].

The present findings further yield potential clinical implications. As children diagnosed with SAD did not show a negative evaluation bias regarding their gaze behavior, some often-implemented CBT strategies aimed at correcting negative self-evaluations, for example, cognitive restructuring or video feedback, might not be appropriate for all affected children. For those children showing visual avoidance of faces, a behaviorally oriented therapy component should initially be implemented [46]. It is, however, important to note that we were not able to distinguish between a social skills deficit regarding visual attention to faces and anxiety-induced performance deficits [47]. To improve individually tailored therapy interventions, research is needed to examine developmental trajectories regarding visual attention, especially considering the pronounced variability of gaze behavior found in our sample, indicating that late childhood may be an interim phase during which stable visual attention patterns emerge [9].

The current results must be interpreted in light of certain limitations. Although the mobile eye-tracking glasses enhanced the ecological validity of our results considerably, they also resulted in a reduced spatial resolution compared to stationary eye-tracking measures and prevented, for example, a more fine-grained spatial analysis of eye contact. As eye-tracking glasses are further highly salient to the wearer, they may have drawn attention to gaze behavior, which may have influenced our results. A further limitation is that only one social context, a highly structured social stress task with explicit performance requirements, was utilized. While this approach allowed us to implement a comparable social situation for all participants, task demands, such as cognitive load (e.g., [48]), may have influenced gaze behavior. Future studies should consider comparing social attention in children with SAD in different social contexts (e.g., [49]) and including interaction partners displaying a variety of emotional or social cues (e.g., [50]). This might help enhance our knowledge of how attentional processes interact with behavioral inputs in children diagnosed with SAD, for example, early hypervigilance toward negative social feedback followed by later avoidance (cf. hypervigilance–avoidance hypothesis; e.g., [51]).

Despite these limitations, this study is an important addition to the burgeoning eye-tracking literature regarding visual social attention in children diagnosed with SAD. Our findings suggest that assessing visual attention to faces during an in vivo stressor relevant to real-world functioning may be associated with differential visual attention patterns when compared with studies implementing briefly presented static facial stimuli. Although our results suggest a tendency to avoid visual attention to faces in children diagnosed with SAD during a social performance situation, further research is needed to replicate these preliminary results and to gain a differential understanding of visual social attention in real-life social contexts in childhood SAD.

## Summary

Although the visual avoidance of social information is a maintenance mechanism in theoretical models of SAD, research on visual attention during real-life social situations in children diagnosed with SAD is yet missing. The present study measured visual attention to faces of interaction partners (fixation count, dwell time) during a real-life social performance situation using mobile eye-tracking glasses in children aged 9 to 13 years diagnosed with SAD (*n* = 25) and a HC group (*n* = 30). Compared to the HC group, children diagnosed with SAD showed more visual avoidance (i.e., fewer fixations) of the faces of interaction partners during the second but not the first session of the social stress task. While visual avoidance in HC children decreased with declining state anxiety from the first to the second session of the social stress task, no such effect was found in the group diagnosed with SAD. Further, only in the HC group but not in children diagnosed with SAD did subjective anxiety during the social stress tasks statistically predict lower visual attention to the faces of the interaction partners. These results may indicate a stable dysfunctional gaze pattern of visual avoidance unrelated to state anxiety in children diagnosed with SAD during real-life social interactions. No evidence of a negative self-evaluation bias regarding gaze behavior in children diagnosed with SAD was found, yielding potential clinical implications regarding the need to implement behaviorally oriented therapy components alongside strategies aimed at correcting negative self-evaluations in CBT. Although our results suggest alterations in gaze behavior (i.e., a tendency to avoid visual attention to faces) in children diagnosed with SAD during a real-life social performance situation, further research is needed to replicate our preliminary findings, especially considering the medium sample size and the highly structured social performance situation realized in this study.

### Supplementary Information

Below is the link to the electronic supplementary material.

Supplementary file1 (DOCX 952 KB)

## Footnotes

1 Data collection was part of a comprehensive laboratory study evaluating rumination and associated psychophysiological processes in childhood SAD that included the implementation of a cognitive distraction condition. Including the experimental condition as a control variable influenced none of the here-presented results significantly, all *p*s ≥ 0.104. Condition is thus not further included in our data analysis.

2 Preliminary data analysis further indicated that including the control variables age, gender and state anxiety did not improve model fit significantly, all *p*s ≥ 0.401.
