# Supplementary material for: Assessing Visual Avoidance of Faces During Real-Life Social Stress in Children with Social Anxiety Disorder: A Mobile Eye-Tracking Study
Source: Child Psychiatry Hum Dev. 2022 Jun 16;55(1):24–35. doi: 10.1007/s10578-022-01383-y (PMC10796484; doi:10.1007/s10578-022-01383-y)
Supplement: Supplementary file 1 — Supplementary file1 (DOCX 952 KB) [file 10578_2022_1383_MOESM1_ESM.docx]

**Supplementary Material**

Assessing Visual Avoidance Of Faces During Real-Life Social Stress in Children With Social Anxiety Disorder: A Mobile Eye-Tracking Study

**Supplement A**

**Figure A.1**

*Screenshot of an example video scene depicting the area of interest “Face”*

**
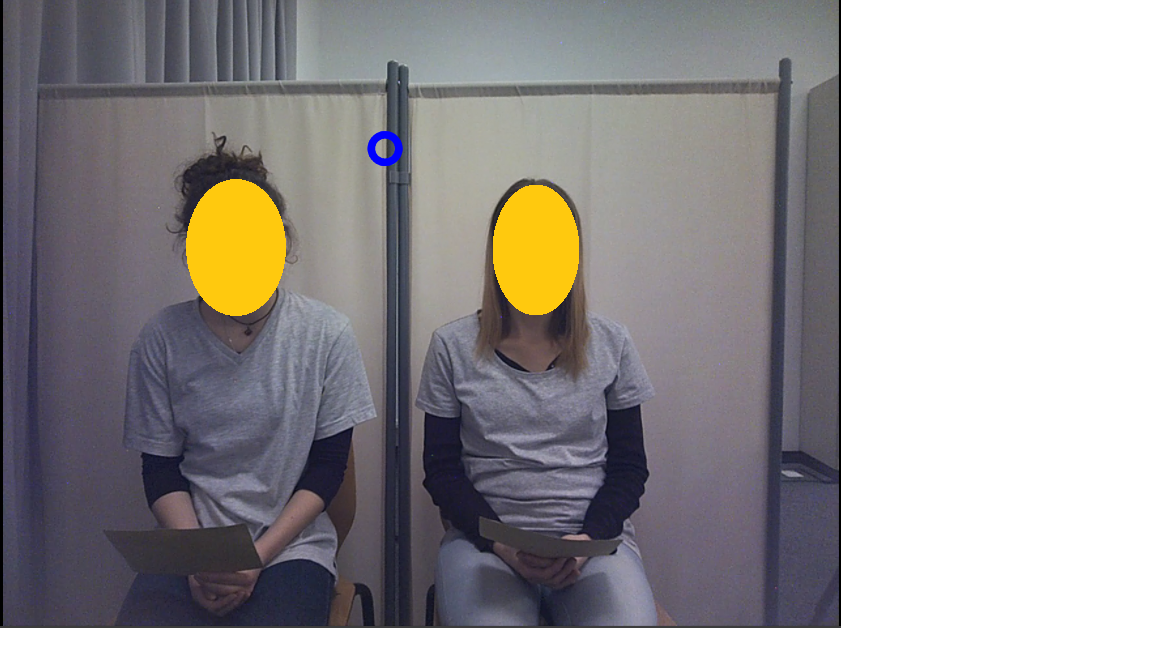
**

*Note.* This figure is a screenshot of a video scene taken during the social stress task depicting the participant’s point of view. Participants were seated opposite two female observers at a distance of 1.20 m. Fixations (here indicated by the blue circle) were manually mapped onto an abstract template by two independent raters, who were blind to group status, using SensoMotoric Instruments’ (Teltow, Germany) standard software (BeGaze 3.7). The areas that were coded as “face area of interest” in the abstract template are marked in yellow. Fixations on the faces of both observers were mapped onto a single template. All observers were asked to wear gray t-shirts or dress themselves modestly to reduce possible influences on gaze behavior.
